# Disseminating research in rural Yup'ik communities: challenges and ethical considerations in moving from discovery to intervention development

**DOI:** 10.3402/ijch.v72i0.20958

**Published:** 2013-08-05

**Authors:** Inna Rivkin, Joseph Trimble, Ellen D. S. Lopez, Samuel Johnson, Eliza Orr, James Allen

**Affiliations:** 1 Department of Psychology and Center for Alaska Native Health Research, University of Alaska Fairbanks, Fairbanks, AK, USA; 2 Department of Psychology, Western Washington University, Bellingham, WA, USA; 3 Center for Alaska Native Health Research, UAF-UAA Joint Ph.D. Program in Clinical-Community Psychology, University of Alaska Fairbanks, Fairbanks, AK, USA; 4 Center for Alaska Native Health Research, University of Alaska Fairbanks, Fairbanks, AK, USA; 5 Biobehavioral Health and Population Sciences, University of Minnesota Medical School, Duluth, MN, USA

**Keywords:** Alaska Native, stress, coping, reporting research results, community-based participatory research (CBPR), research ethics

## Abstract

**Background:**

The native people of Alaska have experienced historical trauma and rapid changes in culture and lifestyle patterns. As a consequence, these populations shoulder a disproportionately high burden of psychological stress. The Yup'ik Experiences of Stress and Coping project originated from rural Yup'ik communities’ concerns about stress and its effects on health. It aimed to understand the stressful experiences that affect Yup'ik communities, to identify coping strategies used to deal with these stressors and to inform culturally responsive interventions.

**Objectives:**

Here, we examine the process of moving from research (gaining understanding) to disseminating project findings to translation into intervention priorities. We highlight the importance of community participation and discuss challenges encountered, strategies to address these challenges and ethical considerations for responsible intervention research with indigenous communities that reflect their unique historical and current socio-cultural realities.

**Design:**

Community-wide presentations and discussions of research findings on stress and coping were followed by smaller Community Planning Group meetings. During these meetings, community members contextualized project findings and discussed implications for interventions. This process placed priority on community expertise in interpreting findings and translating results and community priorities into grant applications focused on intervention development and evaluation.

**Results:**

Challenges included translation between English and Yup'ik, funding limitations and uncertainties, and the long timelines involved in moving from formative research to intervention in the face of urgent and evolving community needs. The lack of congruence between institutional and community worldviews in the intervention research enterprise highlights the need for “principled cultural sensitivity”.

**Conclusions:**

Cultural sensitivity requires sharing results that have practical value, communicating openly, planning for sustainability and incorporating indigenous knowledge and expertise through a community-guided process. Our research findings will inform continued work within our partnership as we co-develop culturally based strategies for multilevel community interventions to address stress.

The native people of Alaska have experienced historical trauma and on-going rapid, often externally imposed changes in culture and lifestyle patterns (1–3). As a consequence, these populations shoulder a disproportionately high burden of psychological stress. Yup'ik communities in the Yukon Kuskokwim Delta region in Southwest Alaska have experienced epidemics and forced acculturation, contributing to behavioural health issues, including substance abuse and suicide (3–5). Cultural loss in Yup'ik communities has resulted in generational gaps that disrupt the transmission of cultural traditions and values important for well-being (6). Despite these intrusions, Yup'ik communities have retained cultural traditions which act as protective factors against the development of physical and psychological illness (7). These cultural protective factors can be harnessed to collaboratively develop culturally grounded interventions that reduce stress and build connections across generations, helping communities move towards wellness on their own terms.

The Yup'ik Experiences of Stress and Coping project originated from rural Yup'ik communities’ expressed concerns about stress and its effects on health. The project aims to better understand the stressful experiences that affect individuals in Yup'ik communities, coping strategies used to deal with them and the role of traditional cultural practices in coping. The goal now is to use project findings to inform culturally responsive interventions. The project is a collaboration between the Center for Alaska Native Health Research (CANHR) at the University of Alaska Fairbanks (UAF), the tribal owned regional Yukon-Kuskokwim Health Corporation (YKHC) and two Yup'ik communities. A community planning group (CPG) in each community guides the research process and comprises people from a variety of backgrounds and community structures, including the tribal office, clinic, school and church. The CPG meetings typically included 10–15 community members, with an evolving membership to add relevant perspectives. The CPGs include men and women of various age groups ranging from young adults in their 20s up to Elders in their 80s.

Translational research applies knowledge gained in research to health-promoting practice and interventions (8, 9). The translational continuum offers a useful framework that guides the long-term planning entailed in health disparities research. The Yup'ik Stress and Coping project focuses on the first phase of the translational cycle, the discovery phase, and early community dissemination efforts are critical to translating research findings into the second translational phase, that of intervention.

## Importance of collaborative community dissemination

The partnership adhered to a Community-Based Participatory Research (CBPR) approach (10), in which the communities guided all aspects of the research, including the research questions, the interview adaptation process, the sampling and recruitment procedures and the schedule of visits to the communities. Collaboration continued as an important element in dissemination of project findings to the community and other stakeholders.

Community dissemination of findings is inherent to the CBPR process. It facilitates co-learning, enhances the validity of the research, builds trust, strengthens partnerships, empowers communities and guides priorities for future interventions (11). Allotment of adequate time and resources for disseminating findings to the community (11, 12) was an important consideration for the Yup'ik Stress and Coping project. This included extensive efforts to develop presentations that were culturally appropriate, helpful and understandable to community audiences through the involvement of cultural consultants and community members in the community dissemination planning process (11–13).

## Ethical considerations

Reporting results back to communities is not only important to intervention development but also an ethical responsibility. Increasingly, this responsibility has been incorporated into ethical guidelines for researchers, including guidelines for research with indigenous populations in the circumpolar north, which emphasize the importance of using clear non-technical language to report findings responsive to local concerns, and translating these findings into the languages of those affected (14, 15). Decisions about the dissemination process have ethical implications and can determine if diverse perspectives are represented in discussions of the findings, and if lessons learned are applied in future dissemination (11).

Dissemination of findings to collaborating communities helps community members determine the relevance of findings to their values and needs, guide the directions for future community-based work and make decisions regarding broader dissemination. Community and tribal entities are increasingly involved in such decisions (16, 17). This is a question of who owns the data and has decision-making authority on how findings are handled. Increasingly, indigenous communities claim data ownership. From this perspective, the community has the final decision about dissemination of results outside the community; this requires close involvement of community members with any research effort. Effective dissemination in indigenous communities not only increases the likelihood that the findings presented are accurate and free of cultural misunderstandings but also conveys the message that researchers recognize communities as capable of understanding the implications of research findings and of resolving local issues.

## Objective

Here, we examine the process of moving from conducting community-based assessment of priority needs and resources to the dissemination of findings for translation into viable community-guided interventions. Particular emphasis is placed on the role of community participation in interpreting project findings, and moving from understanding to intervention. We describe challenges encountered, strategies to address these challenges and ethical considerations for responsible research with indigenous communities that reflect their unique historical and socio-cultural realities.

## Design

Methodology of the Yup'ik Stress and Coping project, including interview procedures and the community–academic partnership, are described in a previously published manuscript (18). The partnering communities, YKHC Human Subjects Committee and UAF Institutional Review Board, approved the research protocol and dissemination. Here, we focus on the process for disseminating findings back to the partnering communities.

### Collaborative dissemination process

The CANHR team (located in Fairbanks, in Alaska's interior region) made several visits to the two Yup'ik communities (located in Southwest Alaska) to discuss emerging findings from their interviews about stress and coping. During each visit, findings were presented through community-wide dissemination meetings in the tribal community center. Smaller meetings with the CPG followed these public presentations. Yup'ik language translation of presentations and meetings was provided by Eliza Orr, a professional translator and Yup'ik cultural consultant, and/or by a community member hired as a field research assistant.

**Fig. 1:**
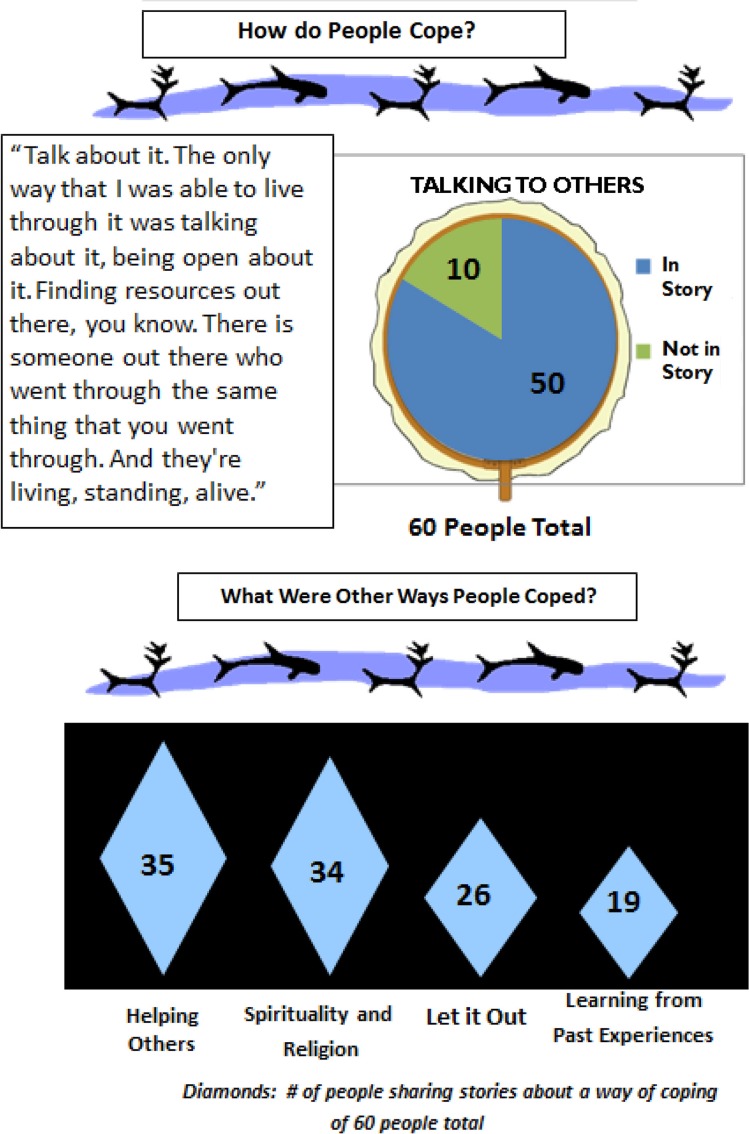
Example of charts for culturally appropriate dissemination.

Previous CANHR experiences suggested that conventional bar charts and numerical comparisons were not easily understandable, struck people as foreign and unfamiliar, and were not the best method for engaging Yup'ik communities (12). These experiences informed the development of more culturally conducive dissemination materials. Data were presented in graphs using locally relevant symbols, such as the eye-catching diamond shapes that adorn traditional parkas instead of bars in graphs, and sections of hand drums instead of pie charts (see Fig. 1).

On the basis of community preference determined by the CPG, findings in one community were presented in Yup'ik (with PowerPoint slides in English), while in the other, findings were presented in English with simultaneous translation into Yup'ik. Dissemination procedures incorporated local dialect and vocabulary preferences, using examples of concepts for which there was no Yup'ik equivalent (12, 13), and local photographs whenever possible to visually describe concepts. Printouts of all dissemination materials were provided. During and after the presentations, the community members were encouraged to ask questions and to provide their thoughts and feedback. They were invited to contact the research team with thoughts that might come to mind later, to allow people to reflect on findings, leaving open the door for additional feedback. This is important because being thoughtful about what one says and taking time to think before one speaks is highly valued within Yup'ik culture: “Words have power to change, transform, heal and harm; they must be used very carefully” pg. 139 (5). Yup'ik values also include learning through observation and contemplation, as well as experience and guidance from Elders (4, 5). These values were guiding principles throughout the research collaboration forming the current project.

Meetings with the CPG followed community dissemination presentations. CPG meetings began with an opening prayer led by an Elder, followed by an invitation for CPG members to share comments about the findings, how they could be useful to the community and how they could inform intervention development. CPG members also discussed how various funding opportunities fit with community priorities and provided guidance on grant applications. Elders were then invited to share any additional thoughts and guidance and to lead the closing prayer for each meeting.

During the presentations and CPG meetings, community members further contextualized the project findings. They discussed how the findings fit with their experiences, the changes they had seen in the community and the continuing struggles. Participants talked about the cultural meaning of findings, such as how these coping strategies fit with the Yup'ik way of life, with Elders’ teachings and how coping has changed through the generations. They weighed implications of the findings for intervention priorities and activities. For example, they emphasized how interventions should build upon the cultural and community strengths identified in the research. They noted how different activities were appropriate based upon the season of the year. They emphasized the importance of the activities in connecting people across generations, and in particular, in involving Elders in teaching cultural values and healthy ways of coping. Community members also suggested additional analyses. For example, one community asked to see findings specific to their community, along with findings that combined data across both communities, to best inform intervention activities that fit their local context. Other analysis requests included looking at differences across age and gender. The upshot of this intensive process with the CPG and broader community was to apply their community expertise to interpretation of findings to facilitate the translation into plans for further intervention research and grant applications.

## Results

### Challenges

Challenges associated with translation and differences between Yup'ik and academic worldviews emerged during the dissemination process. These challenges illustrate a number of broader tensions academic and community partnerships face as they seek to build shared understanding and move towards intervention.

#### Translation of meaning across languages

Translation between Yup'ik and English was critical throughout the study, including during dissemination of findings. Elders whose primary language was Yup'ik were able to participate in the interviews and the dissemination process. This allowed a rich and meaningful exchange of culturally grounded knowledge. This was especially critical given the traditional value of respect for Elders which guides Yup'ik social norms (4, 5, 19).

However, translation also posed challenges illustrating deeper issues for intervention science. Often, key concepts in the research did not directly translate between Yup'ik and English. For example, there is no single word for “stress” in Yup'ik. The closest concept in Yup'ik is umyuaq caknerluni, translating as “trouble in the mind”. This potentially poses barriers for cross-cultural understanding and collaborative intervention development; it also informs the project regarding complexity and nuance in local understandings.

We were fortunate enough to have skilled and experienced translators on our team. Before meetings, translators devised several strategies for describing difficult concepts. One strategy involved providing specific local examples for difficult to translate terms. For example, stress might be described as when you have too much to do (caarkalissiiyaalleq), when your kids are distracting you (irniavet tuavvluten) or when something is bothering you and you are worried (umyuarniurluni). In other cases, several alternative phrasings were provided. Translators also consulted with local community bi-lingual Elders or family members for suggestions on how to translate or describe specific words.

#### Diverse worldviews

These language differences often surfaced deeper underlying differences between the values and worldviews of the communities, and the perspectives and practices of Western science, which affect the intervention research enterprise (20, 21). For example, funding agencies typically require interventions to focus on a specific disease and be broadly generalizeable beyond the community of interest in the study. In contrast, the two partnering communities desired holistic, strength-based interventions focused on general well-being, as understood from within the frame of their distinct cultural context. Community partners expressed discomfort with many of the procedures of Western science, at times noting their incongruity with local cultural values. For example, withholding interventions from communities as part of a randomized controlled trial conflicts with values of sharing and events being open to all.

#### Differences between research timelines and community needs

We also confronted funding limitations and logistical challenges associated with conducting research in remote communities. Weather and transportation interruptions provided further uncertainties, along with our shared commitment to respect practices that stop work around funerals, deaths, major subsistence activities and other community events. These protracted research timelines were frustrating in the face of urgent community needs. The formative research and community assessment required to build understanding can take years. The grant writing and review process for continued funding extends timelines. The principles of CBPR include a focus on translating knowledge into intervention and social action (10, 11, 22). Tension can form when prolonged research and funding trajectories in the current tight and uncertain funding climate interfere with the timely delivery of intervention to address community needs. The lengthy timelines required to move from research to applying findings for useful community intervention warrant ethical considerations regarding risk and benefit, beginning with the potential for demoralization of communities who engage in formative research that then goes no further when intervention development is unfunded.

### Strategies to address challenges and ethical considerations

A number of strategies assisted our partnership in addressing these challenges to move from understanding to intervention.

#### Sharing findings that are useful

One important strategy was to disseminate findings that were useful and practical. Thus, even before the intervention phase, the research findings could be used to benefit community members. This commitment was forefront as we prepared community presentations. We also integrated input from community members to make findings more useful by, for example, making them more specific to their community. We also discussed interim products community members would like to see from our work together, which included, for example, a coping workshop and a youth–Elder storytelling event.

#### Communication

Open communication and constantly gauging expectations vis-à-vis these challenges was vital to our partnership. On-going community conversations helped build awareness about the limitations, uncertainties and requirements of the research and funding process. We maintained a commitment to the communities but were careful to refrain from making promises about future activities that we could not keep, given reliance on uncertain external funding. It was important for the team to convey realistic expectations about the extremely competitive nature of grant funding, and the time required in preparing a competitive grant application and the review process. Our conversations with key community leaders emphasized the importance of careful planning that can take months to nurture and develop.

These community discussions increased the academic partners’ awareness of community priorities and resources. This emphasized the responsibility of the university researchers for translating institutional funder perspectives to community members so that they could make informed decisions, and the responsibility of university researchers to push for adjustments, whenever possible, in the dissemination process and design of future intervention research to fit the Yup'ik way. For example, although the planned intervention focuses on a specific intervention target (Yup'ik young adults), other generations would be included in intergenerational mentoring activities, consistent with community values of passing knowledge across generations. Although randomized trials conflict with values of sharing benefits, interventions would be tested using innovative designs consistent with community values.

#### Planning for sustainability

Planning for sustainability is another critical ethical priority, particularly within a climate of uncertain funding. One important element of this is fostering community capacity to mount efforts on their own, regardless of the ability to obtain continued external funding. Community conversations regarding future interventions illuminated the importance of integrating the intervention into on-going community structures and activities. In the CPG meetings, partners discussed strategies for sustainability during a gap in funding, while also working towards funding for the subsequent phases of the translational research enterprise.

#### Integrating cultural knowledge

Community discussions focused on the importance of integrating local cultural understanding into dissemination and intervention. This involved utilizing cultural knowledge from Elders to contextualize findings and inform intervention activities. For example, during a CPG meeting, community members discussed the importance of narrative stories (including personal, historical, and mythical stories) for passing on cultural values and knowledge of how to cope. CPG members then organized a story-telling night, in which Elders shared their cultural knowledge through stories recorded by community members. In another community visit, presentations on project findings regarding the importance of role models and preparation to prevent stress sparked CPG discussions of cultural strengths inherent to the community, including cultural values about preparing for things ahead of time, and passing on knowledge to younger generations. In CPG meetings in another community, discussion of findings on the importance of family for coping sparked conversations about community activities that brought families together and how these helped prevent substance abuse in young people. The CPG then worked with other community structures to organize a coping workshop integrating project findings, which included speakers discussing coping, respect, hope, one's inter-relationships with others, wisdom and cultural knowledge, with Yup'ik dances interspersed between the speakers. We also presented project findings at a CANHR translational health workshop in Bethel (the regional hub), in which partners from many Yup'ik communities and members of the YKHC regional health corporation shared input on findings and discussed the application of cultural practices such as coming together as a community. Community members also highlighted cultural practices and values, such as the importance of potlatches, the value of subsistence activities, the significance of language and the role of Elders in passing on cultural knowledge, in a conference presentation in which they were co-presenters.

## Conclusions

The project experiences with community dissemination illustrate the importance of collaboration and the value added through the dissemination and ensuing community discussions. The collaborative dissemination process is transformational, helping to catalyze change in both local and scientific understanding. This process informs science by engaging community perspectives on findings, contextualizing results and integrating cultural knowledge. This community expertise also molds subsequent dissemination and intervention work, ensuring it fits community priorities and benefits the communities.

Despite these benefits, it is also important to recognize the difficulties of CBPR, and its interface with procedures that may not fit with community worldviews and an unstable funding structure. Conducting CBPR in the context of these uncertainties and extensive timelines can weigh heavily on collaborators who have forged trusting long-term relationships. It is critical to maintain open communication regarding these benefits and challenges in partnering communities as well as in academic arenas.

The literature describing the process of community dissemination and challenges faced in dissemination work with remote arctic communities is sparse. Yet dissemination is integral to the CBPR process. It requires additional consideration, along with time and resources built into CBPR grants to allow for giving back findings, engaging community members and gathering community perspectives. Research should investigate effective strategies for community dissemination (11–13) and creative routes for fostering community members’ involvement (23).

Community dissemination addresses limitations of conventional scientific reports. Technical reports are rarely used at the local level because the writing is often not accessible to community members and the reports typically do not provide direct input to immediate decisions communities face. Without more accessible dissemination efforts, the community can be left with the feeling that the research was a wasted effort, and they may develop a negative attitude towards research in general. Thus, dissemination efforts should use non-technical language to specifically address local needs (14, 24). Community dissemination also informs scientific dissemination, when the context community members can provide is integrated into findings, ensuring accurate interpretation in light of local culture, values or beliefs (25).

Research with populations facing health disparities should create opportunities for improved services. One means to improve the quality of services is the cultural adaptation of interventions to make them more acceptable, improve their cultural fit and increase their utility in real-world settings. Such cultural adaptation must respect communities’ self-determination rights and integrate their understandings of both the problem and solutions (26). Interventions are likely to be more effective when they are congruent with the culture and context of the person (27). Yet, how culture plays a role in the process of interventions and their adaptation to meet the needs of diverse individuals and populations is still a challenge for the field (28). It is similarly important to recognize research itself constitutes an intervention that can have various and at times unintended effects on the participants and the community in which it is done. Community dissemination helps build the bridge from the discovery to intervention phases of the translational continuum of health disparities research (8).

Community dissemination has a significant role in ensuring responsible and ethical research with indigenous populations to address health disparities. Collaborative dissemination builds partnerships, contextualizes findings and guides priorities for future intervention. When done effectively, it facilitates integration of indigenous knowledge and expertise through a community-guided process, enriching the research findings and providing a bridge for moving from understanding to intervention. The current project experiences illustrate the importance of dissemination that is timely, understandable and helpful, along with persistence through obstacles and commitment to a long-term view of partnership.
